# The long non-coding RNA AK001796 contributes to tumor growth via regulating expression of p53 in esophageal squamous cell carcinoma

**DOI:** 10.1186/s12935-018-0537-8

**Published:** 2018-03-16

**Authors:** Bin Liu, Chun-Feng Pan, Guo-Liang Yao, Ke Wei, Yang Xia, Yi-Jiang Chen

**Affiliations:** Department of Thoracic and Cardiovascular Surgery, The First Affiliated Hospital of Nanjing Medical University, 300 Guangzhou Road, Nanjing, 210029 Jiangsu China

**Keywords:** Esophageal squamous cell carcinoma, lncRNA AK001796, Tumor growth, Tumorigenicity, MDM2/p53 signaling pathway

## Abstract

**Background:**

Esophageal squamous cell carcinoma (ESCC) is one of the prevalent and deadly cancers worldwide, especially in China. Considering the poor prognosis of ESCC, the aim of this study is to dissect the effects of long non-coding RNA (lncRNA) AK001796 on cell proliferation and cell cycle in vitro and tumorigenicity in vivo, providing therapeutic targets for ESCC.

**Methods:**

We conducted quantitative real time PCR to detect the expression level of lncRNA AK001796 in human ESCC tumor and adjacent non-tumor tissues, and analyzed the correlation between lncRNA AK001796 expression and clinicopathologic feature of ESCC patients. Then we knocked down the expression of lncRNA AK001796 in human ESCC cell lines Eca-109 and TE-1, and next inspected cell cycle and apoptosis condition in these cells using flow cytometry. Subsequently, we used CCK-8 assay to test proliferation ability of the lncRNA AK001796-silenced ESCC cells, and the MDM2/p53 signaling pathway in these cells was analyzed by western blot analysis. At last, the ESCC xenograft models were established to verify the role of lncRNA AK001796 on the occurrence and development of ESCC.

**Results:**

In this study, we demonstrated that lncRNA AK001796 was significantly upregulated in ESCC tumor tissues compared to adjacent non-tumor tissues. Knockdown of lncRNA AK001796 inhibited ESCC cell growth, cell cycle, and tumor growth in a xenograft mouse model via regulating MDM2/p53 signal pathway. The expression of lncRNA AK001796 was positively correlated with MDM2 levels in human ESCC samples.

**Conclusions:**

Overall, lncRNA AK001796 regulates cell proliferation and cell cycle via modulating MDM2/p53 signaling in ESCC, which provides a new insight into the treatment targets for ESCC.

*Trial registration* This study was registrated in the Ethics Committee of the First Affiliated Hospital of Nanjing Medical University (Trial registration: 2012-SR-127, Registered 20 January 2012)

**Electronic supplementary material:**

The online version of this article (10.1186/s12935-018-0537-8) contains supplementary material, which is available to authorized users.

## Background

Esophageal carcinoma ranks seventh in cancer incidence and sixth in cancer-related death worldwide, 90% of which is esophageal squamous cell carcinoma (ESCC) [1, 2], and the 5-year survival rate of advanced stage of ESCC is only 10% [3]. With its high occurrence rate and mortality, a better understanding of the molecular mechanisms underlying ESCC is urgently needed for improving the survival rate of ESCC.

Long non-coding RNAs (lncRNAs) are transcripts longer than 200 bp nucleotides and appear to lack of protein-coding capacity on chromosome 12 [4, 5] (Additional file 1: Figure S1). Recent years, just as the siRNAs and microRNAs, the lncRNAs have emerged as a novel class of important regulators of gene expression and attracted increasing attention in the field of cancer [6], such as lung cancer [7], bladder cancer [8], gastric cancer [9], colorectal cancer [10], ESCC [11] and so on. Though numerous lncRNAs have been identified, there were a good deal of biological functions that still remain unknown [12]. As a member of lncRNAs, lncRNA AK001796 was reported to promote cell growth and tumorigenicity in lung cancer [13], but its fuction in ESCC is undiscovered till now, and our study is to explore its potential influence in ESCC.

P53, as one of the primary “gatekeepers” of the cell and a tumor suppressor gene, is activated in responding and sensing to a variety of stresses, and facilitates induction of cell cycle and apoptosis [14]. Mouse double minute 2 (MDM2), an E3 ubiquitin ligase, has been identified as a crucial downregulator of p53, which could inhibit transcriptional activity of p53, promote its ubiquitination and degradation [15–17]. The MDM2/p53 is one of the important signaling pathways, which regulates some radical cellular activities, for example, cell growth and cell cycle [18]. However, the regulatory role of lncRNA AK001796/MDM2/p53 signaling in ESCC is unknown.

In our study, we detected the expression level of lncRNA AK001796 in ESCC tumor tissues and paracarcinoma tissues, and investigated its relationship with clinicopathologic characteristics in ESCC. Then, tumor inhibition of lncRNA AK001796 knockdown was explored in vitro and in vivo. Moreover, we also demonstrated the regulation of lncRNA AK001796 in MDM2/p53 signaling pathway. Our findings indicated that lncRNA AK001796 played a vital role in the progression of ESCC, and our study may provide a new insight into the treatment targets for ESCC.

## Methods

### Patients and tissue samples

All samples from fresh ESCC and paracarcinoma tissues were obtained from patients with ESCC who received radical esophagectomy in the First Affiliated Hospital of Nanjing Medical University. Then all the tumor samples were collected immediately and frozen stored at − 80 °C. All tumor specimens and paired normal tissues were confirmed by experienced pathologists. The clinical and pathological characteristics of each patient were also collected. All procedures involving the use of human tissues were approved by the Ethics Committee of the First Affiliated Hospital of Nanjing Medical University (Ethical Application Ref: 2012-SR-127) and all had been performed in accordance with the Helsinki Declaration, every patient signed the informed consent before our study.

### Cell culture

Human ESCC cell lines Eca-109 and TE-1 were purchased from American Type Culture Collection (ATCC, USA). Eca-109 and TE-1 cells were grown in RPMI-DMEM containing high glucose (GIBICOBRL) and supplemented with 10% fetal bovine serum (BI, Australia). All cells were cultured in an incubator at 37 °C with 5% CO_2_.

### RNA extraction and quantitative real time PCR

The total RNA of tissues and cells were extracted by using ReliaPrepTM RNA Miniprep System (Millipore, USA), according to the manufacturer’s instructions, then reverse-transcribed the RNA into first strand cDNA using the HiFiScript 1st Strand cDNA Synthesis Kit (CWBIO, China). Quantitative real time PCR was performed with the SYBR Premix Ex Taq™ Kit (TaKaRa, Japan) in a 20 μl reaction system. 18S rRNA was used as an internal control. The primers of lncRNA AK001796 and 18S rRNA were as follows: lncRNA AK001796 sense, 5′-GCCCAGAUUUAAGGGCUAUTT-3′; and reverse, 5′-AUAGCCCUUAAAUCUGGGCTT-3′; 18S rRNA sense, 5′-GTAACCCGTTGAACCCCATT-3′; and reverse: 5′-CCATCCAATCGGTAGTAGCG-3′. Reactions were performed on a Roter-Gene 6000 (Corbett Research, Sydney, Australia) according to the following conditions: initial denaturation 30 s at 95 °C, followed by 40 cycles at 95 °C for 5 s and 60 °C for 30 s. The data were analyzed with the 2^−ΔΔCt^ method, normalized to 18S rRNA levels. All primers were designed and synthesized by Beijing Genomics Institute (China), each experiment was performed three times.

### RNA interference

Four different siRNAs targeting lncRNA AK001796 and the siRNA control which we referenced the article [13] were designed and synthesized by GenePharma (Shanghai, China). The siRNA molecules were double-stranded RNA oligonucleotides with proprietary chemical modifications. The freeze-dried siRNAs were dissolved in RNase-free water and stored at − 20 °C. The sequences of the four siRNAs and the siRNA control were as follows:

siRNA-1 Forward: 5′-GGUCACUACUGCUUUAUAATT-3′,

Reverse: 5′-UUAUAAAGCAGUAGUGACCTT-3′;

siRNA-2 Forward: 5′-GGUGGCCUGUACCUAUAAUTT-3′,

Reverse: 5′-AUUAUAGGUACAGGCCACCTT-3′;

siRNA-3 Forward: 5′-GCCCAGAUUUAAGGGCUAUTT-3′,

Reverse: 5′-AUAGCCCUUAAAUCUGGGCTT-3′;

siRNA-NC Forward: 5′-UUCUCCGAACGUGUCACGUTT-3′,

Reverse: 5′-ACGUGACACGUUCGGAGAATT-3′.

### Cell transfection

The Eca-109 and TE-1 cells were trypsinized and grown in six-well plates. All cells were transfected with the siRNAs and the siRNA-NC respectively using Lipofectamine™ 2000 (Invitrogen, shanghai, China) when cell confluence came to 50%. The RNAi duplexes (40 nM) were diluted in 200 μl of serum-free medium and mixed gently. Lipofectamine (4 μl) was then added to another 200 μl of serum-free medium. After 5 min, these two mediums were mixed and incubated for 15 min at room temperature before transfection. The RNAi duplex-Lipofectamine complexes were added to each well and mixed gently by rocking the plate. Cells were harvested after 24 or 48 h of the transfection for qRT-PCR or western blot analysis respectively.

### Western blot analysis

The total proteins from cells were collected with RIPA buffer (Sangon Biotech, Shanghai, China). Cells were collected from a 6-well plate using 100 μl of RIPA buffer per well. The lysates were homogenized and then centrifuged at 16,000*g*. Proteins from the supernatant were quantified by a BCA assay (Thermo, USA), then added an equal volume of 1 × loading buffer and then denatured for 5 min at 100 °C. A total of 20 μg of protein from each sample was separated on a 10% SDS-PAGE gel and transferred onto a PVDF membrane (Bio-Rad). The membranes were blocked for 1 h at room temperature in 5% fat-free milk in Tris-buffered saline with 0.1% Tween-20, and then were incubated overnight at 4 °C with the following primary antibodies: anti-p53 (1:4000, Abcam, USA), anti-p21 (1:2000, Cell Signaling Technology, USA), anti-MDM2 (1:2000, Cell Signaling Technology, USA) and anti-GAPDH (1:10,000). Then, the membranes were incubated with the corresponding secondary antibodies at 1:10,000 for 1 h at room temperature. The immune complexes were examined by ECL detection (Millipore, USA). For quantification, the western blotting bands were quantified by ImageJ software (National Institutes of Health).

### Cell cycle and apoptosis analysis

The apoptotic analysis of Eca-109 and TE-1 cells were stained with Annexin V-FITC and propidium iodide 48 h after transfection and performed using a flow cytometer (FACScan; BD Biosciences, USA). Cells were classified as viable, dead, early apoptotic, or apoptotic. The percentage of early apoptotic cells was analysed by FlowJo 7.6.

For cell cycle analysis, cells were stained with propidium iodide using the BD Cycletest Plus DNA Reagent Kit (BD Biosciences, USA). The relative ratio of cells in G0/G1, S, or G2/M phase was analysed by FlowJo 7.6. Each experiment was performed three times.

### Cell counting kit-8 assay

Cells proliferation was detected by cell counting kit-8 (CCK8) assay (Promega, USA) after the cells transfected with siRNAs, according to the manufacturer’s protocol. The transfected cells were grown in 96-well plates (2000 cells/well), then added 10 μl CCK8 solution to 90 μl DMEM medium and incubated for 3 h then measured the absorbance at 450 nm. The absorbance was measured at 12, 24, 48 and 72 h after cells were transfected with siRNA-AK001796 respectively.

### Tumorigenicity assays

Five-week-old Balb/c nude mice were housed in specific pathogen-free conditions. All experimental procedures involving animals were in accordance with experimental animal center of the First Affiliated Hospital of Nanjing Medical University and the Ethical Committee of the First Affiliated Hospital of Nanjing Medical University. All animal experiments protocol were conducted according to the animal care ethical guidelines of the Review Committee for the Use of Human or Animal Subjects of the First Affiliated Hospital of Nanjing Medical University. Samples (0.2 ml) of culture medium containing 5 × 10^6^ siRNA-3 or siRNA-NC transfected Eca-109 cells were injected subcutaneously into the opposite posterior flanks of each same mouse (total six mice and three mic per group). Tumor growth was examined every 3 days. The result was monitored by the length (L) and width (W) of the tumor, then calculated the tumor volume (V) according to the formula (L × W^2^ × 0.5, L means length and W means width). Half a month later, the mice were euthanized, the tumors were harvested and the weight of tumors was measured.

### Statistical analysis

Experiments were performed in triplicate at least. Statistical analyses were performed with SPSS 16.0 software. Other numerical data were reported as the mean ± S.E.M. Two-tailed Student’s t test was used to evaluate the difference in protein expression and mRNA levels. A value of P less than 0.05 was considered statistically significant.

## Results

### Overexpression of lncRNA AK001796 in esophageal squamous cell cancer tissues

**Fig. 1:**
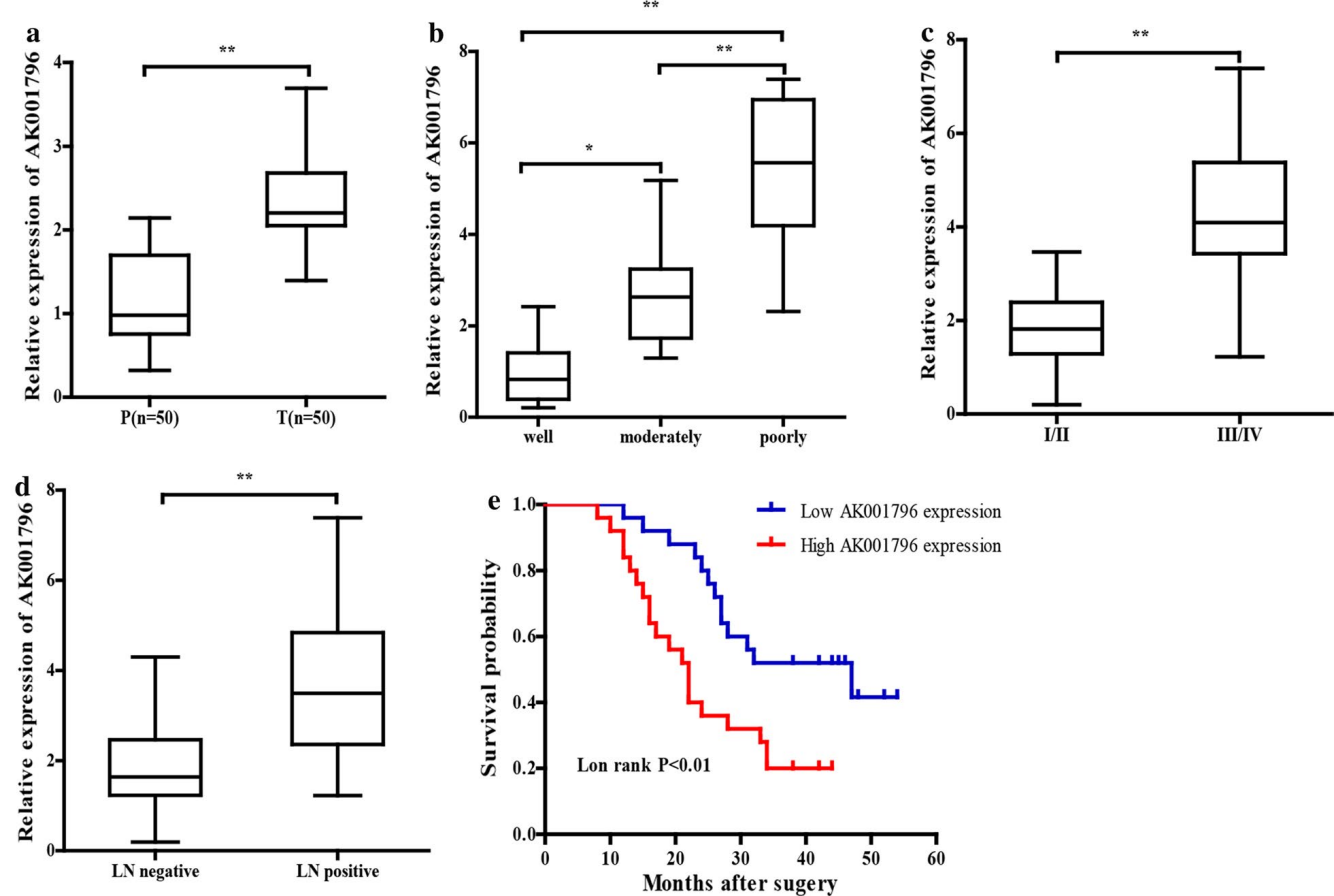
The overexpression of AK001796 in esophageal squamous cell carcinoma and paracarcinoma tissues. **a** qRT-PCR technology detect the mRNA level of lncRNA AK001796 in 50 pairs of esophageal squamous cell carcinoma and paracarcinoma tissues; **b** the mRNA expression of lncRNA AK001796 in different tumor differentiation; **c** the mRNA expression of lncRNA AK001796 in different TNM degrees; **d** the mRNA expression of lncRNA AK001796 with different LN metastasis; **e** the survival curve in lncRNA AK001796 high expression group and low expression group (*LN* lymph node)

The expression of lncRNA AK001796 was detected by qRT-PCR in 50 pairs of ESCC and paracarcinoma tissues. LncRNA AK001796 was significantly overexpressed in ESCC tissues compared with the paired paracarcinoma tissues on the mRNA level (Fig. 1a). Correlation between the expression level of lncRNA AK001796 and clinicopathologic characteristics was further analyzed, and showed that the expression of lncRNA AK001796 were positively related with the degree of tumor differentiation, tumor TNM stage, tumor size and lymph node metastasis (Table 1). High level of lncRNA AK001796 was significantly associated with high differentiation (Fig. 1b), advanced TNM stages (Fig. 1c), and lymph node (LN) metastasis (Fig. 1d). However, the expression level of lncRNA AK001796 was not significantly correlated with tumor location, smoking history, age, gender or alcohol (Table 1). Then survival analysis was performed to analyze the relationship between the expression level of lncRNA AK001796 and survival time. The 50 patients were classified into two groups according to lncRNA AK001796 expression level. The survival analyses showed that patients with high expression of lncRNA AK001796 had significantly shorter survival time than those with low expression level of AK001796 (Fig. 1e). These evidences proved that lncRNA AK001796 may play a crucial role in ESCC’s development and progression.

**Table 1:** LncRNA AK001796 expression and clinicopathologic characteristics in ESCC tissues

Characteristics	Case number	AK001796 expression		*P* value
		Low	High	
Gender				0.185
Male	38	21	17	
Female	12	4	8	
Age (year)				0.225
< 60	17	6	10	
≥ 60	33	19	15	
Smoking status				0.382
Ever and current	31	14	17	
Never	19	11	8	
Alcohol consumption				0.208
Ever and current	37	16	20	
never	13	9	5	
Histological grade				
Well	12	10	3	0.025*
Moderately	28	13	14	
Poorly	10	2	8	
Tumor location				0.777
Upper 1/3 and middle 1/3	36	13	14	
Lower 1/3	14	12	11	
Tumor stage				0.023*
I/II	28	18	10	
III/IV	22	7	15	
Lymph node metastasis				0.045*
Negative	24	17	9	
Positive	26	8	14	
Tumor size (cm)				0.011*
< 4	30	17	8	
> 4	20	8	17	

*ESCC* esophageal squamous cell carcinoma

* *P *< 0.05

### Knockdown of lncRNA AK001796 regulates cell proliferation and cell cycle in ESCC

**Fig. 2:**
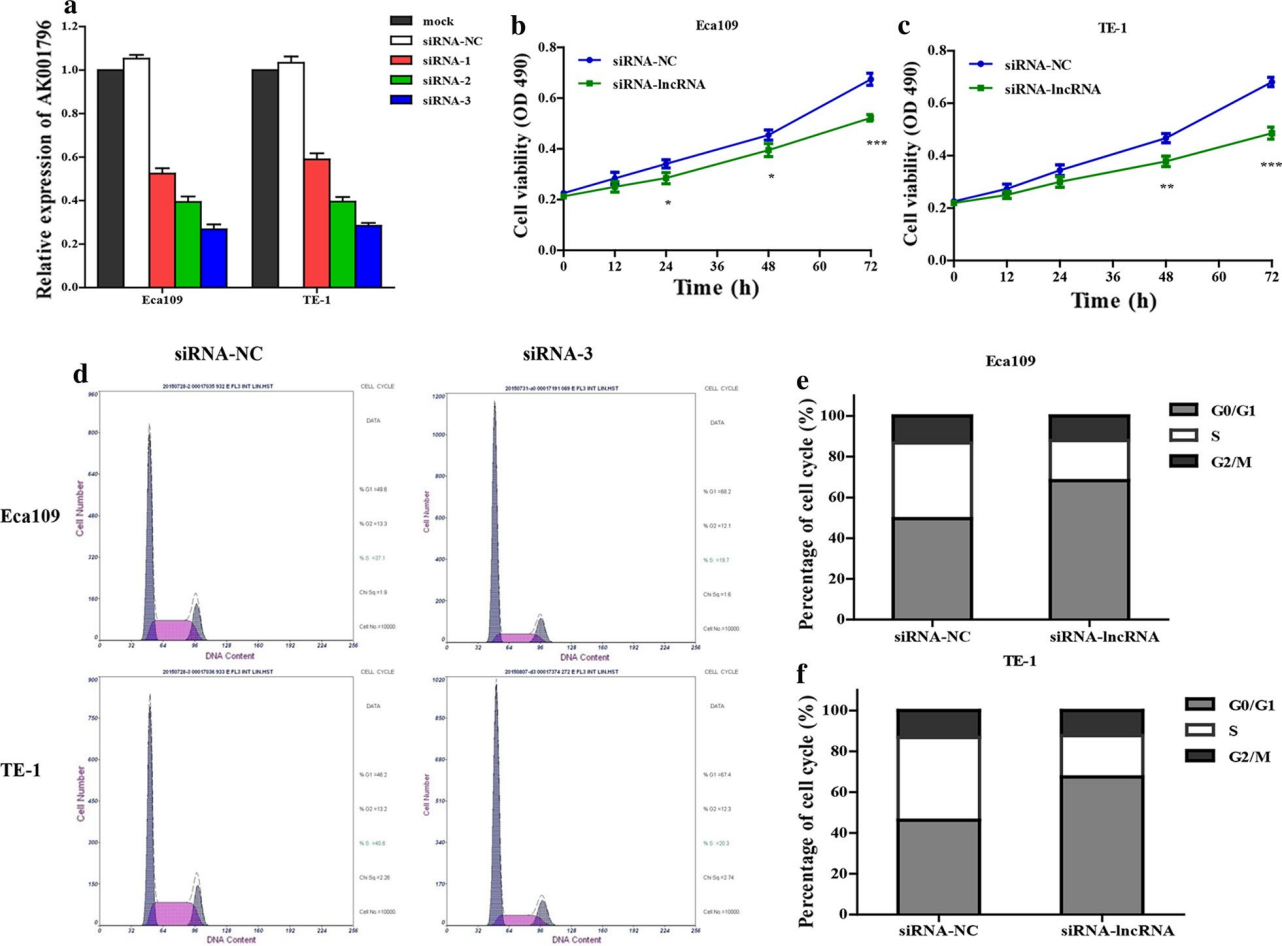
The change of cell proliferation and cell cycle after transfected with siRNA-AK001796 in Ech-109 and TE-1 cell lines. **a** The knockdown efficiency of lncRNA AK001796 in Ech-109 and TE-1 cell lines by transient transfection with lncRNA AK001796 siRNA (1, 2, 3 and NC) was detected by qRT-PCR; **b** and **c** the cell viabilities of Ech-109 (**b**) and TE-1 (**c**) cells were measured by CCK-8 assay; **d** the cell apoptosis was analyzed by flow cytometry; **e** and **f** the cell cycle of Ech-109 (**e**) and TE-1 (**f**) cells was analyzed by flow cytometry (*NC* negative control, n = 3, *P < 0.05, **P < 0.01, ***P < 0.001)

To evaluate the biological role of lncRNA AK001796 in ESCC, siRNAs targeting lncRNA AK001796 was used to suppress the expression of lncRNA AK001796 and demonstrated that the siRNA-3 had the highest knockdown efficiency in both Eca-109 and TE-1 cell lines (Fig. 2a), so we chose the siRNA-3 for the following experiments. Cell proliferation was measured by using CCK8 assay, and the proliferation of ESCC cells (Eca-109 and TE-1 cells) was significantly inhibited after transfected with siRNA-AK1001796, in a time-dependent manner (Fig. 2b, c). Then, we further analyzed whether cell apoptosis and cell cycle were affected by siRNA-AK001796, and the results indicated that there were no influence of lncRNA AK001796 made in ESCC cells apoptosis (Fig. 2d). Moreover, The group transfected with siRNA-AK001796 showed increased percentage of ESCC cells in the G2/M phase and reduced percentage of ESCC cells in S phase obviously (Fig. 2e, f).

### LncRNA AK001796 regulates MDM2/p53 signaling pathway

**Fig. 3:**
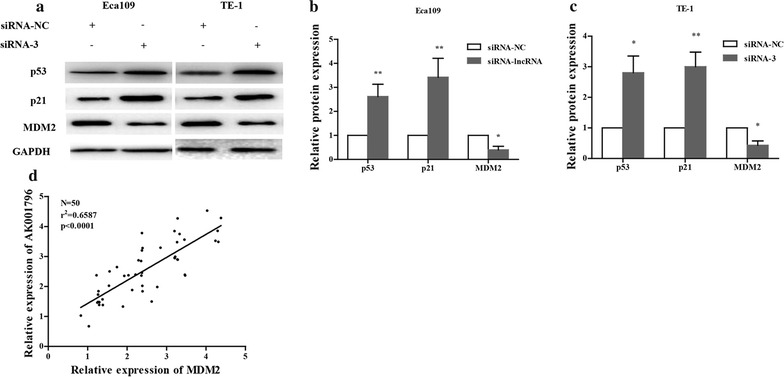
Expression of p53-related genes. **a** MDM2 was downregulated whereas p53 and its target genes p21 were upregulated after transfected with siRNA-AK001796 by western blot (right: mean ± SD, n = 3, *P < 0.05, **P < 0.01); **b** and **c** relative protein expression of p53, p21 and MDM2 which were analyzed by Quantity one 6.0 in Ech-109 (**b**) and TE-1 cells (**c**); **d** correlation analysis between the expression of lncRNA AK001796 and MDM2 (N = 50, r^2^ = 0.6587, *P* < 0.0001)

To determine the potential molecular mechanisms of lncRNA AK001796 in the ESCC cells growth, we futher examined the expression of MDM2/p53 and its target gene p21 by western blot analysis. The results showed that MDM2 was downregulated and the expression of p53 and its target gene p21 were significantly upregulated in both Eca-109 and TE-1 cell lines after transfection with siRNA-AK001796 (Fig. 3a–c). And in the clinical tissues, the expression of lncRNA AK001796 showed a positive correlation with the expression of MDM2 (Fig. 3d). These results suggested that lncRNA AK001797 regulated cell growth and cell cycle via activating MDM2/p53 signaling.

### Knockdown of lncRNA AK001796 impairs the tumorigenicity

**Fig. 4:**
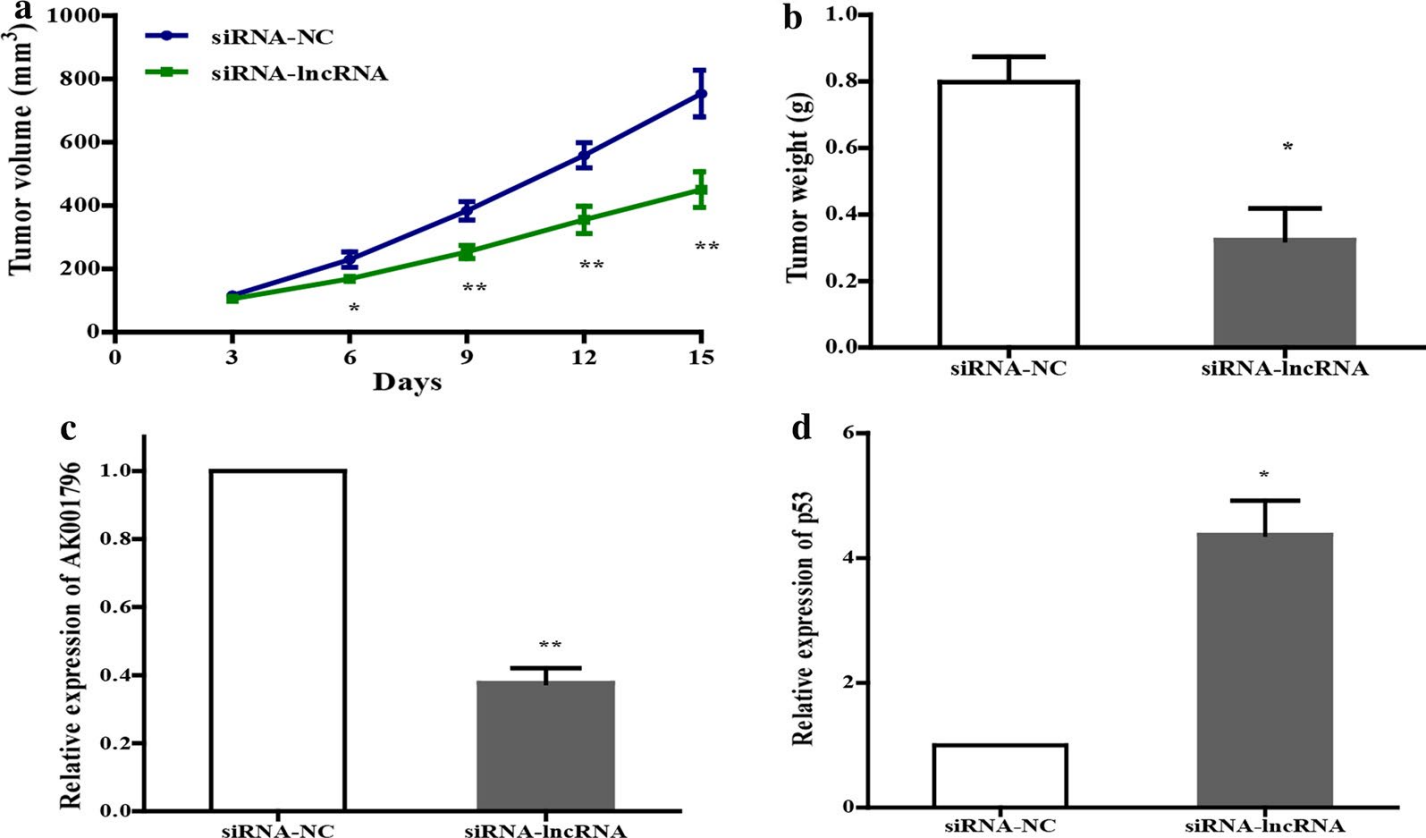
Treated with siRNA-AK001796 impair the tumorigenicity. **a** Xenograft tumors volume derived from siRNA-AK001796 and NC groups and it shows in a time dependent manner; **b** the weight of tumors; **c** the expression of lncRNA AK001796 in the tumor; **d** the expression of p53 in the tumor (mean ± SD, n = 3, *P < 0.05, **P < 0.01)

To further elucidate the effect of lncRNA AK001796 on cancer development, we assessed the impact of lncRNA AK001796 on tumorigenicity in vivo. Eca-109 cells transfected with siRNA-AK001796 were implanted subcutaneously into Balb/c nude mice, observation and measurements were performed every 3 days and total for 15 days after injection. Strikingly, tumor volume was notably reduced in transfected with siRNA-AK001796 group (Fig. 4a). Moreover, in comparison with the control groups, the weight of the tumors in the group treated with siRNA-AK001796 were reduced compared with NC group (Fig. 4b). To further explore the function of lncRNA AK001796, the mRNA levels of lncRNA AK001796 and p53 in the tumors were detected by RT-PCR technology. The mRNA expression of lncRNA AK001796 were decreased in the group treated with siRNA-AK001796 compared with the NC group (Fig. 4c), and the mRNA expression of p53 was increased in the group treated with siRNA-AK001796 compared with the NC group (Fig. 4d).

## Discussion

Esophageal squamous cell carcinoma is the most aggressive malignancies, characterized by high metastasis. In the present study, we evaluated the expression of lncRNA AK001796 in human ESCC tissues by RT-PCR technology. And the results showed that lncRNA AK001796 was highly expressed in tumor tissues, indicating its critical roles in tumorigenesis of ESCC. We demonstrated that level of lncRNA AK001796 also positive related with the patients’ TNM degrees, LN metastasis, tumor size and the tumor differentiation. Besides, lncRNA AK001796 level was negatively correlated with the survival time of patients and could serve as the prognostic factor. All these results demonstrated that lncRNA AK001796 was involved in the progress and development of ESCC.

Recent studies have shown that lncRNAs play pivotal roles in the initiation and progression of ESCC, for example, the lncRNA HOX transcript antisense RNA (HOTAIR) was reported to control cell cycle of ESCC [19]; the lncRNA HOTTIP promotes metastasis and the EMT progression of ESCC [20]; the lncRNA SPRY4-IT1 contributes to increased ESCC cells viability by activating zinc finger 703 expression [21] and so on. And in our study we verified the lncRNA AK001796 can regulate cell growth and cell cycle in ESCC cells, and found that knockdown of lncRNA AK001796 contributed to the G2/M stage arrest both in Ech-109 and TE-1 cell lines.

Mouse double minute 2 has the capacity to be activated by Akt signal pathway, and its activation results in the degeneration of its targets p53, p53 activation can excitate its nuclear target p21 as well [22, 23]. Additionally, MDM2/p53 singling is related to cell growth, cell cycle and cell apoptosis [16, 24, 25], and lncRNAs play an important role in the regulation of cell growth by modulating p53 pathway [26, 27]. In this study, we found that knockdown of lncRNA AK001796 upregulates p53 expression and inhibits MDM2 expression, suggesting that lncRNA AK001796 is involved in the regulation of MDM2/p53 singling on cell cycle and cell proliferation in ESCC cells.

## Conclusions

In conclusion, we found that upregulation of lncRNA AK001796 was a common event in ESCC and high expression of lncRNA AK001796 could indicate the poor prognosis of ESCC patients. Knockdown of lncRNA AK001796 could inhibit ESCC cell proliferation and induce the cell cycle arrest in G0/G1 phrase by regulating MDM2/p53 signaling. We propose that lncRNA AK001796/MDM2/p53 could serve as an effective therapeutic target and potential prognostic factor for ESCC patients.

## Additional file

**Additional file 1: Figure S1.** Schedule of AK001796 location in chromosome 2.

## Abbreviations

ESCC: esophageal squamous cell carcinoma
lncRNA: long non-coding RNA
MDM2: mouse double minute 2
ATCC: American Type Culture Collection
GAPDH: glyceraldehyde-phosphate dehydrogenase
DMEM: dulbecco’s modified eagle medium
LN: lymph node
