# Supplementary figures and images for: The long non-coding RNA AK001796 contributes to tumor growth via regulating expression of p53 in esophageal squamous cell carcinoma
Source: Cancer Cell Int. 2018 Mar 16;18:38. doi: 10.1186/s12935-018-0537-8 (PMC5857070; doi:10.1186/s12935-018-0537-8)

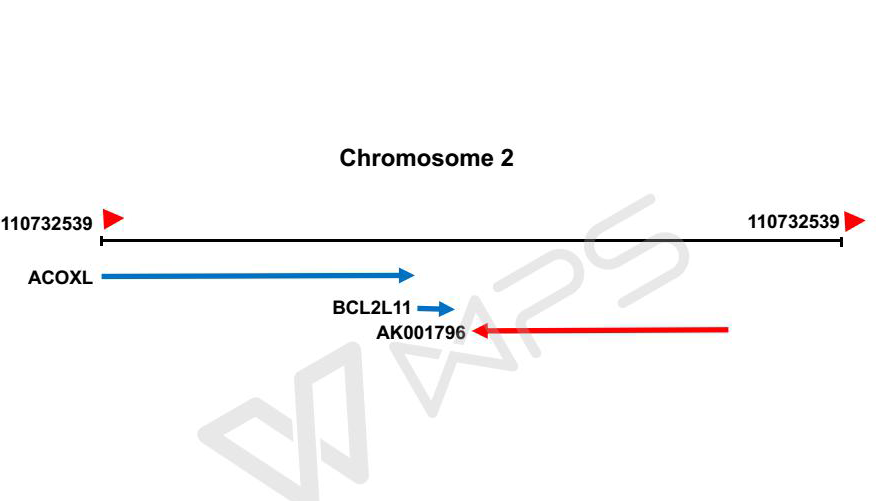

Supplement: Supplementary file 1 — Additional file 1: Figure S1. Schedule of AK001796 location in chromosome 2. [file 12935_2018_537_MOESM1_ESM.tif]
